# Dynamic structure driven image scrambling technique for data protection

**DOI:** 10.1038/s41598-025-28925-3

**Published:** 2025-12-21

**Authors:** Swapan Kumar Shee, Jyoti Khandelwal, Amit Kumar Bairwa

**Affiliations:** 1 Department of Computer Science & Engineering, University of Engineering & Management, Jaipur, 303807 India; 2 Department of Computer Science & Engineering, Amity University Rajasthan, Jaipur, 303002 India; 3 Department of Artificial Intelligence and Machine Learning, School of Computer Science and Engineering, Manipal University Jaipur, Jaipur, 303007 India

**Keywords:** Engineering, Mathematics and computing

## Abstract

Today, communication using digital media has increased rapidly. In this digital communication era, providing security to sensitive images is essential at the time of transmission. The images are mostly focused nowadays because they are used directly or indirectly in every field of information sharing, for example, healthcare, military, intellectual property, and many more areas. In this paper a new approach to image scrambling is proposed to secure the sensitive image information. The proposed method is focused on the core concept of data structure. It involves the use of binary trees and the efficiency of hash tables, which enhances image security during transmission. The dynamic data structure properties enhanced the scrambling and descrambling process. In the scrambling method, the image pixels are first stored in the binary tree using the hash table. After the binary tree arrangement, pixels are collected into a one-dimensional array using the tree traversing process. Now, the one-dimensional array is converted into the two-dimensional array to match the size of the original image. The descrambling method is the inverse of the scrambling method. The proposed method maintains the quality of the image for both sender and receiver; different quality assessment parameters like PSNR, MSE, NCC, AD, SC, MD, Corr, HC, VC, DC, NPCR, UACI and Entropy are used to check the outcome. The outcome of PSNR between the original image and the scrambled image is less than 4 dB. For the descrambled image and the original image, the PSNR is infinite. According to the obtained results, there is a 100% similarity between the original image and the descrambled image. The proposed method was also compared with the existing methods, and it showed a negative or near to ‘0’ correlation between the scrambled image and the original image. In future work the proposed scrambling method can be used in image watermarking or image steganography techniques.

## Introduction

In the past few years, working habits have changed in terms of communication, shopping and transaction techniques. As a result, a lot of visual content is exchanged on the media and on networks. This type of transmission can lead to hacking, copyright infringement, and unauthorized access. Visual data related to the medical, military and government sectors is very secret. To ensure the safety and security of the data, different techniques are used, such as cryptography, steganography, encryption, watermarking, etc. To protect the content of the visual data, research has been carried out over the years.

**Fig. 1:**
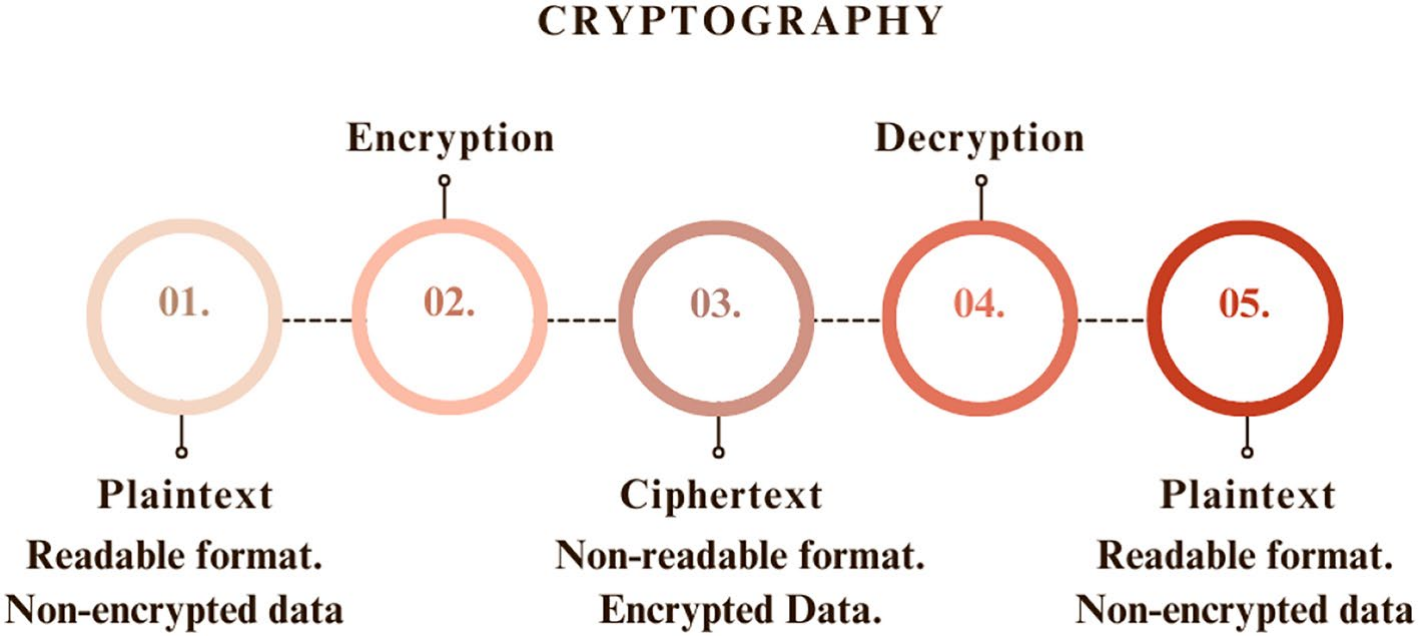
General block diagram of cryptography.

Cryptography^1^ is a study that is used to ensure the security of images during multimedia transmission. In order to hide the original content of the visual data, plaintext has been converted to ciphertext, which is not readable and understandable by third parties. In the decryption phase, the ciphertext will be decrypted to its original content using different algorithms and keys. The working of cryptography is shown in Fig. 1.

**Fig. 2:**
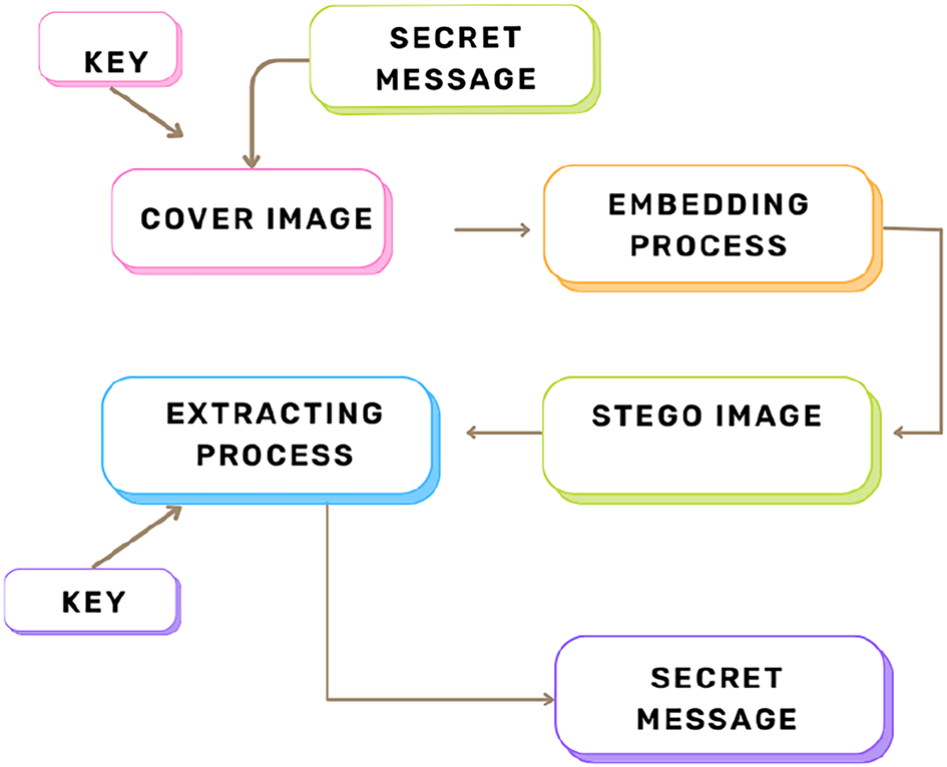
General block diagram of steganography.

Steganography^2^ is another method of image security that has been increasingly used in recent years. In this method, secret messages are embedded within the cover image and the resultant image is called the stego image. In the decoding phase, using the stego image and keys, the secret original message can be revealed. In recent years, interest in steganography has shifted from traditional and ancient practices to hiding secret data and media objects^3^, especially secret image files, in image files^4^. The mechanism of Steganography is shown through a block diagram in Fig. 2.

**Fig. 3:**
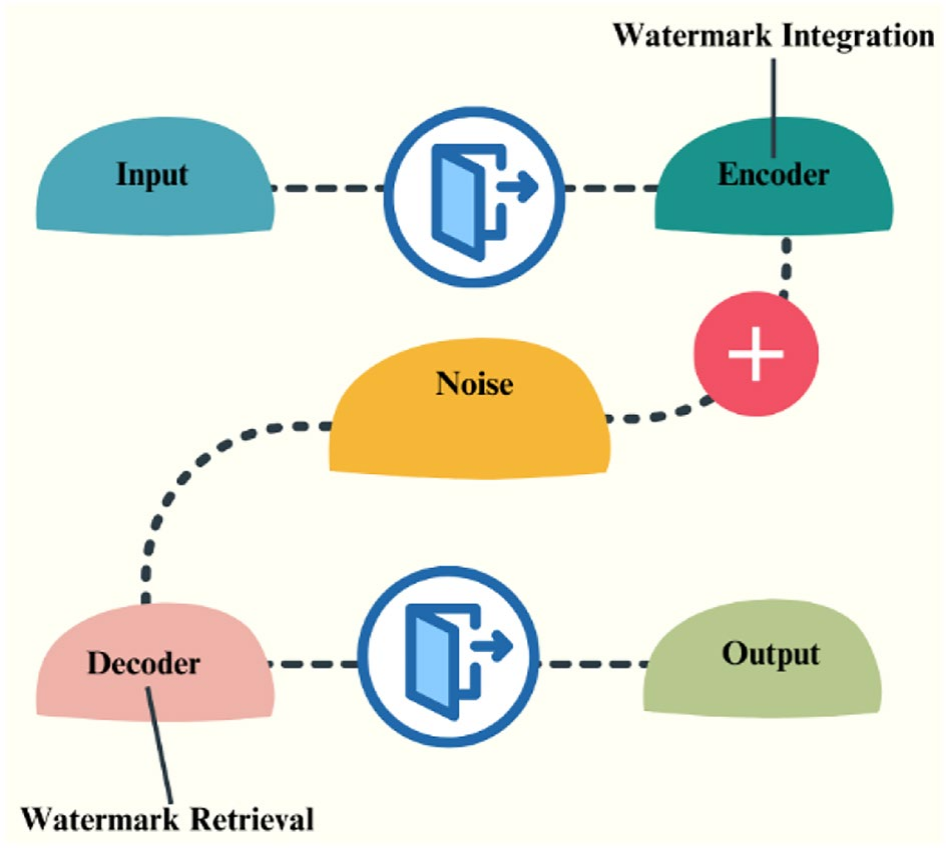
General block diagram of watermarking.

Watermarking^5^ is a technique similar to steganography. In watermarking, the visual content will be encoded with the watermark integration and the noise. Watermarks can be visible (e.g., overlaying text or logo) or invisible (embedded within the content). On the receiver end, the encoded image can be decoded by removing the watermarking. The framework of the watermarking system is represented by a block diagram in Fig. 3.

A 2023 analysis by Cybersecurity Ventures estimates that by 2025, cybercrime will cost the global economy $10.5 trillion yearly, with digital picture manipulation and data breaches accounting for a sizable amount of this total. Particularly, industries such as healthcare and defence are more vulnerable to image tampering, where unapproved changes might have catastrophic repercussions. The creation of safe and reversible picture scrambling techniques to ensure data integrity and secrecy during transmission is highly motivated by these growing concerns.

In this paper, an efficient image scrambling and descrambling method is proposed that provides high security for image transmission through multimedia. The use of dynamic data structures, i.e., binary trees and hash tables, leads to the high efficiency of the proposed method. This paper is structured in different sections. Section 1, i.e., “Introduction,” consists of a discussion about cryptography, steganography, and watermarking techniques. Some image security-related work has been discussed in Section 2, i.e., “Literature Review.” In Section 3, i.e., “Proposed Method,” the proposed scrambling and descrambling methods, followed by their algorithms, have been presented. Section 4, i.e., “Experimental Results,” consists of an experimental setup, and experimental results from the proposed method have been discussed. And the last section, Section 5, i.e., “Comparative Study,” consists of a comparison between different image security techniques and the proposed method based on different performance measure metrics.

The proposed method differentiates itself from existing image scrambling techniques by introducing a novel, structure-aware approach based on data structures such as binary trees and hash tables. Although most traditional techniques rely on fixed transformations, permutation matrices, or chaotic maps, our method takes advantage of the logical organization and dynamic properties of these data structures to enhance both security and retrieval accuracy.

## Literature review

In this section, the required terminologies related to image scrambling and descrambling methods are discussed. Also, the section highlighting the previously developed scrambling methods.

### History of scrambling methods

As current threads on image security are increasing, this opens up new research fields for researchers in this domain. This section discusses some of the previously completed work, where image scrambling techniques can be broadly divided into structural scrambling (^6,7^), chaotic scrambling (^8,9^), and hybrid techniques (^10,11^). References are discussed in detail to provide a better understanding of each approach, and several articles that compare different scrambling methods across these categories are also reviewed (^12–15^).

In^6^ a comparative study has been performed between different scrambling techniques such as 2D mapping, bit plane scrambling, matrix transformation, and key-based row and column shifting techniques. As a result, it is revealed that matrix-based image scrambling applied to the Arnold transform is performing better than other techniques. In^7^ (steganography and cryptography), two techniques have been executed that help secure transmission. This includes the techniques of hiding the text in the image using the LSB (Least-significant bit) algorithm, image inside the image using the least significant bit and genetic algorithm, video steganography using hamming code, steganography using cryptography, and deep neural networks.

In^8^ different chaotic maps depending on different scrambling methods and performance measures have been discussed. Scrambling methods include the Fibonacci-Lucas sequence, the logistic map, the Baker map, the Henon map, the Arnold map, etc. And performance measures like NPCR (Number of Pixels Change Rate), UACI (Unified Average Changing Intensity), mean value analysis, entropy, and peak signal-noise ratio are measured. In^9^ three different cryptographic techniques, such as Arnold Transform, Rivest Code 4 (RC4), and one-time pad (OTP), have been implemented, and performance measures like UACI, SSIM (structural similarity index measure), NPCR, entropy, computation time, and histogram analysis have also been calculated for comparison. Three techniques have their own advantages, like Arnold transform has an advantage on histogram, RC4 has an advantage on entropy value, and OTP has an advantage on SSIM and UACI.

In^10^ the use of DWT (Discrete Wavelet Transform) in different steganography techniques and its algorithm have been discussed. Image steganography techniques based on DWT include Haar DWT, diamond encoding in DWT, redundant DWT, QR factorization, DWT, and the Data Mining ID3 (Iterative Dichotomiser 3) algorithm. In^11^ two image watermarking techniques have been discussed using DCT, DWT, and SVD. The first is a hybrid watermarking technique using DCT-DWT-SVD, and the second is a conventional DCT-DWT-SVD hybrid watermarking technique with an image scrambling method (Arnold transform). For the validation of techniques, normalized correlation (NC) has been executed, and as a result, it was found that the second one is performing better.

In^12^ image encryption techniques based on 2D chaotic maps like Arnold 2D cat map, Baker map, Henon map, cross chaos map, and 2D logistic map have been studied. To check the efficiency of every technique, NPCR (Number of Pixels Change Rate) and UACI (Unified Average Changing Intensity) values have been calculated, and a comparison has been implemented. As a result of the comparison, NPCR is around 99%. The experimental result shows that the Arnold 2D cat map is better than other chaotic maps. In^13^ different digital steganography techniques have been discussed, and for validation, mean square error (MSE) and peak signal-to-noise ratio (PSNR) have been calculated. The techniques include Mod 10, DCT, PVD (pixel value differencing) 2, PVD 1, Stream of 1s and 0s, IP (Inverted Pattern Approach), and OPAP (Optimum Pixel Adjustment Procedure).

In^14^ a comparative analysis of different scrambling methods and their parameters has been executed. Analysis consists of two methods: one is queue transformation, and another is logistic mapping using chaotic sequence scrambling. As a result, SSIM for the logistic transform is less than the queue transform, which makes transmission safer. In^15^ various CA-based DIS techniques with the same parameters have been implemented. CA-based DIS techniques have been analyzed based on varying image sizes. The robustness of all the techniques has been validated using correlation coefficient analysis and the number of pixels change rate. In “A novel DNA-based key scrambling technique for image encryption,” an efficient DNA-based key scrambling method has been proposed. The proposed method has been evaluated using 15 different datasets, and performance has been measured based on entropy, key space, cipher pixel correlations, variance of the histogram, time complexity, and PSNR.

### Overview of famous scrambling methods

Some Famous scrambling methods has been discussed in Table 1, which provides an edge in the era of image security. Table 1 is designed to give an overview of the performance and characteristics of each method based on these features, which are key for determining the appropriate use case and the level of security or efficiency needed.

**Table 1:** Comparative study table focusing on the famous scrambling methods with mathematical details.

Feature/method	Arnold’s transformation^16^	Fibonacci transformation^17^	Gray code bit plane^18^	2D mapping	Row and column shifting	Quantum scrambling^19^	Circular shifting	Pixel swapping^20^	Rubik’s cube^21^
Complexity	Moderate to High	Low to Moderate	Moderate	Low to Moderate	Low	High	Low	Moderate	High
Key Sensitivity	High	Moderate	Moderate	Low	Low	High	Moderate	Moderate	High
Computational Efficiency	Moderate	High	Moderate	High	High	Moderate	High	Moderate	High
Resistance to Cryptanalysis	High	Moderate	Low	Moderate	Moderate	High	Moderate	Moderate	High
Ease of Implementation	Moderate	Easy	Moderate	Easy	Easy	Moderate	Easy	Moderate	Complex
Image Quality Preservation	Moderate to Low	High	High	High	High	High	High	High	Moderate
Scalability	High	Low	Moderate	High	High	Moderate	High	High	High
Application Areas	Secure image encryption	Simple encryption and watermarking	Image encryption and data hiding	General image manipulation	Basic image scrambling and data hiding	Advanced cryptographic applications	Simple image scrambling and data manipulation	Basic data hiding and image manipulation	Advanced scrambling and puzzle-based applications
Mathematical Basis	Permutation matrix: $X_{\text {new}} = A X_{\text {old}} A^{-1}$, where *A* is Arnold matrix	Fibonacci sequence (iterative)	Gray code bit-plane conversion	Coordinate mapping: $(i,j) \rightarrow f(i,j)$	Linear shift: *R*(*i*), *C*(*j*)	Quantum superposition and entanglement	Circular permutation: $p(i) \rightarrow p(i+k)\bmod N$	Swap matrix: $P(i,j) \leftrightarrow P(j,i)$	Group theory and permutation cycles (Rubik’s Cube)

## Proposed methods

**Fig. 4:**
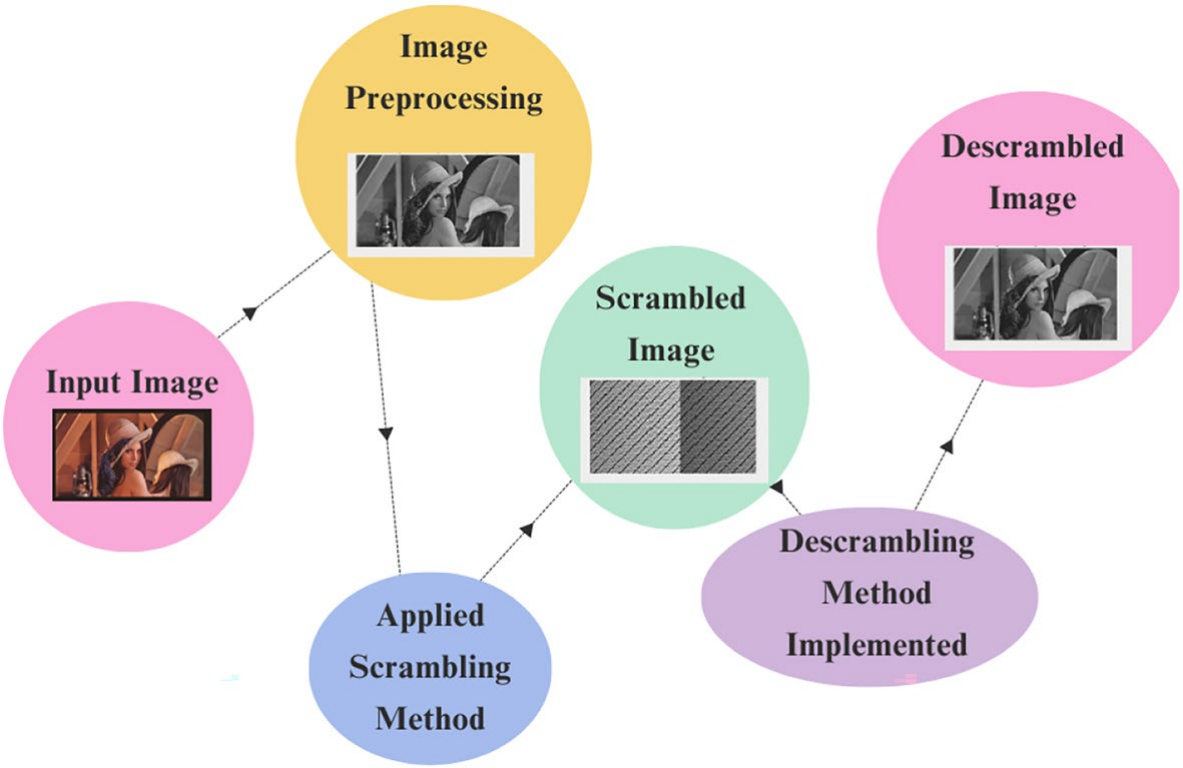
Framework of the proposed method.

The proposed methods are divided into two folds: one is scrambling, and the other is descrambling. The scrambling part involves rearranging the pixels of the original image, which makes the original image more private from unauthorized users. The descrambling part involves reordering the pixel of the scrambled image using the proposed algorithm and keying it to its own original format. The central approach of this proposed method is the integration of dynamic data structures like binary trees and hash tables, which makes the image security technique easier and more efficient. In this section proposed scrambling method and proposed descrambling method has been elucidated through detailed implementation using Proposed Algorithm and Flow Chart. Figure 4, showing the proposed scrambling method framework.

### Proposed scrambling method

**Fig. 5:**
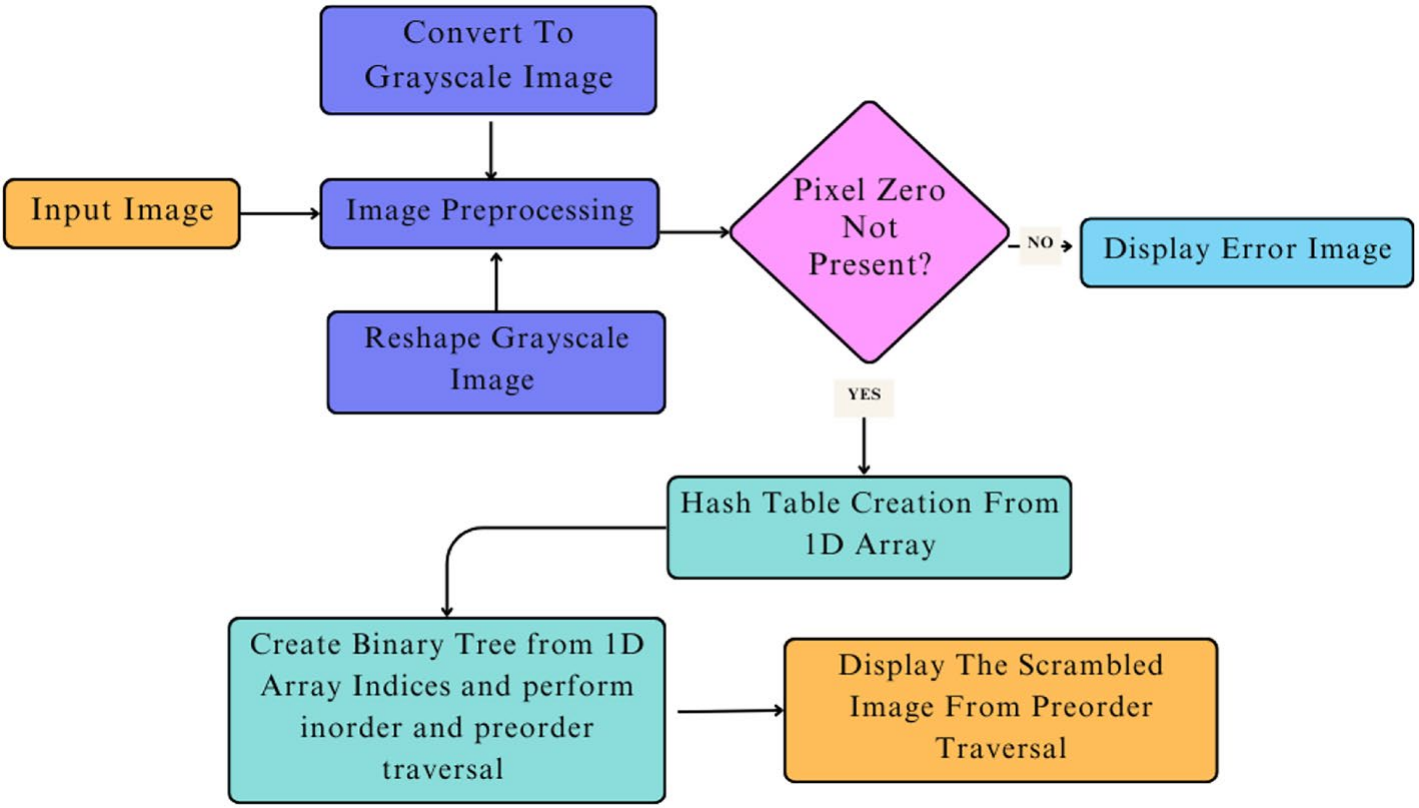
Proposed model for scrambling method.

The proposed scrambling method divides the whole scrambling process into different substages. Starting with the loading of the input image, which may or may not be a color image^22^, the image is preprocessed by converting the input image to a grayscale image if not and reshaping the grayscale 2D image array to a 1D image array. Next, finding the presence of pixel value 0 can be a limitation of the proposed image scrambling method. Continue with hash table creation from the 1D image array along with the creation of binary trees from the 1D image array indices, perform the in-order and pre-order traversals, and return the scrambled image from the pre-order traversal. The framework of the proposed scrambling method is shown in Fig. 5 and described in Algorithm ’1’.

Below is a formal mathematical representation of the hash structure and traversal logic used in proposed image scrambling method, including the hash function, collision handling, and tree traversal are discussed.

#### Image pixel representation

Let an input grayscale image be represented as a 2D matrix:

1 $$\begin{aligned} I = [I(i, j)] \quad \text {where } 0 \le i< H,\; 0 \le j < W \end{aligned}$$

- *H*: height (number of rows)
- *W*: width (number of columns)

Each pixel *I*(*i*, *j*) has an intensity value in the range:

2 $$\begin{aligned} I(i,j) \in [0, 255] \end{aligned}$$

To process the image, flatten it into a 1D array:

3 $$\begin{aligned} P = [p_0, p_1, \dots , p_{N-1}], \quad \text {where } N = H \times W \end{aligned}$$

#### Hash table structure

We define a hash table *H* to store pixel values and their positions:

4 $$\begin{aligned} H[k] = (p_k, i_k, j_k) \end{aligned}$$

To determine the index in the table, we use a hash function:

5 $$\begin{aligned} h(p_k, i_k, j_k) = (a \cdot p_k + b \cdot i_k + c \cdot j_k) \mod M \end{aligned}$$

Where:

- $p_k = I(i_k, j_k)$ is the intensity value at pixel position $(i_k, j_k)$.
- *a*, *b*, *c* are constants (preferably prime numbers) to reduce collisions.
- *M* is the size of the hash table, ideally $M \ge N$.
- *h* returns the hash index for each pixel entry.

#### Collision handling

In case of hash collisions, we use **chaining**, meaning each index in *H* holds a linked list of pixel entries:

6 $$\begin{aligned} H[k] = \{ (p_{k1}, i_{k1}, j_{k1}), (p_{k2}, i_{k2}, j_{k2}), \dots \} \end{aligned}$$

The expected number of collisions using chaining in a well-designed hash table is:

7 $$\begin{aligned} \mathbb {E}[\text {collisions}] = \frac{N}{M} \end{aligned}$$

To minimize collisions, choose *M* such that $M \approx N$ or select a small prime number greater than *N*.

#### Binary tree construction

Once hashed, pixels are inserted into a Binary Search Tree (BST) or a Balanced BST (e.g., AVL Tree), using the hash index as the key:

8 $$\begin{aligned} \text {Insert } H[k] \text { into } T, \quad \text {where } T \text { is the binary tree} \end{aligned}$$

Each node in the binary tree stores the following information:

9 $$\begin{aligned} \text {Node} = (h, (p_k, i_k, j_k)) \end{aligned}$$

Where:

- *h* is the hash index.
- $(p_k, i_k, j_k)$ is the pixel value and its position in the original image.
- The tree *T* maintains order based on *h* to enable efficient traversal and retrieval.

#### Tree traversal for scrambling

To scramble the pixels, perform an **in-order traversal** of the binary tree:

10 $$\begin{aligned} S = \text {InOrder}(T) \end{aligned}$$

Where:

11 $$\begin{aligned} S = [s_0, s_1, \dots , s_{N-1}] \end{aligned}$$

is the one-dimensional scrambled sequence of pixels obtained by visiting nodes in the order: Left Subtree $\rightarrow$ Root Node $\rightarrow$ Right Subtree.

After that, to scramble the pixels using a **pre-order traversal**, apply a recursive traversal function:

12 $$\begin{aligned} S = \text {PreOrder}(T) \end{aligned}$$

Where:

13 $$\begin{aligned} S = [s_0, s_1, \dots , s_{N-1}] \end{aligned}$$

is the one-dimensional scrambled sequence obtained by visiting nodes in the order: Root Node $\rightarrow$ Left Subtree $\rightarrow$ Right Subtree.

Reshape this 1D array *S* into a 2D scrambled image:

14 $$\begin{aligned} I_s = \text {reshape}(S, H, W) \end{aligned}$$

Example: Binary Tree Insertion and Traversal on a 2D Grayscale Image

**Step 1: Input 2D Image (3** $\times$ **3)**

We use a simple $3 \times 3$ grayscale image matrix:

$$\begin{aligned} I = \begin{bmatrix} 34 & 120 & 45 \\ 78 & 255 & 60 \\ 90 & 10 & 200 \\ \end{bmatrix} \end{aligned}$$

**Step 2: Flatten the Image with Coordinates**

**Table:** 

Index	Value	Coordinates (i, j)
0	34	(0, 0)
1	120	(0, 1)
2	45	(0, 2)
3	78	(1, 0)
4	255	(1, 1)
5	60	(1, 2)
6	90	(2, 0)
7	10	(2, 1)
8	200	(2, 2)

**Step 3: Apply Hash Function**

We define the hash function:

15 $$\begin{aligned} h(p, i, j) = (3p + 5i + 7j) \mod 100 \end{aligned}$$

**Table:** 

Value	(i,j)	Hash (mod 100)
34	(0,0)	102 $\rightarrow$ 2
120	(0,1)	367 $\rightarrow$ 67
45	(0,2)	141 $\rightarrow$ 41
78	(1,0)	239 $\rightarrow$ 39
255	(1,1)	782 $\rightarrow$ 82
60	(1,2)	209 $\rightarrow$ 9
90	(2,0)	289 $\rightarrow$ 89
10	(2,1)	83 $\rightarrow$ 83
200	(2,2)	574 $\rightarrow$ 74

**Step 4: Insert into Binary Search Tree (BST)**

Using the hash values as keys, the binary tree is built as follows:

- Insert (2, 34) $$\rightarrow$$ root
- Insert (67, 120) $\rightarrow$ right of 2
- Insert (41, 45) $\rightarrow$ left of 67
- Insert (39, 78) $\rightarrow$ left of 41
- Insert (82, 255) $\rightarrow$ right of 67
- Insert (9, 60) $\rightarrow$ right of 2
- Insert (89, 90) $\rightarrow$ right of 82
- Insert (83, 10) $\rightarrow$ left of 89
- Insert (74, 200) $\rightarrow$ left of 82

Tree structure (in key, value format):


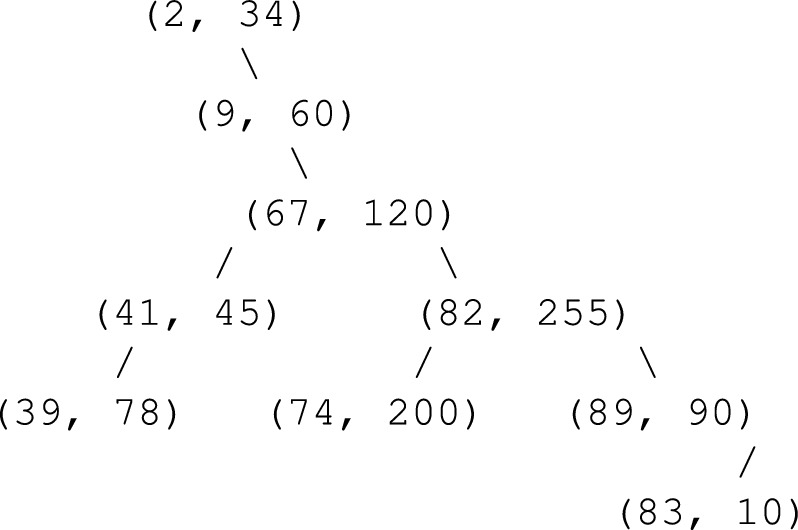


**Step 5: Tree Traversals** **Pre-order Traversal (Root** $\rightarrow$ **Left** $\rightarrow$ **Right):**

Order of visit:

$$(2,34), (9,60), (67,120), (41,45), (39,78), (82,255), (74,200), (89,90), (83,10)$$

**Pre-order Result:**

$$[34,\;60,\;120,\;45,\;78,\;255,\;200,\;90,\;10]$$

**In-order Traversal (Left** $\rightarrow$ **Root** $\rightarrow$ **Right):**

Order of visit:

$$(2,34), (9,60), (39,78), (41,45), (67,120), (74,200), (82,255), (83,10), (89,90)$$

**In-order Result:**

$$[34,\;60,\;78,\;45,\;120,\;200,\;255,\;10,\;90]$$

**Algorithm 1:**
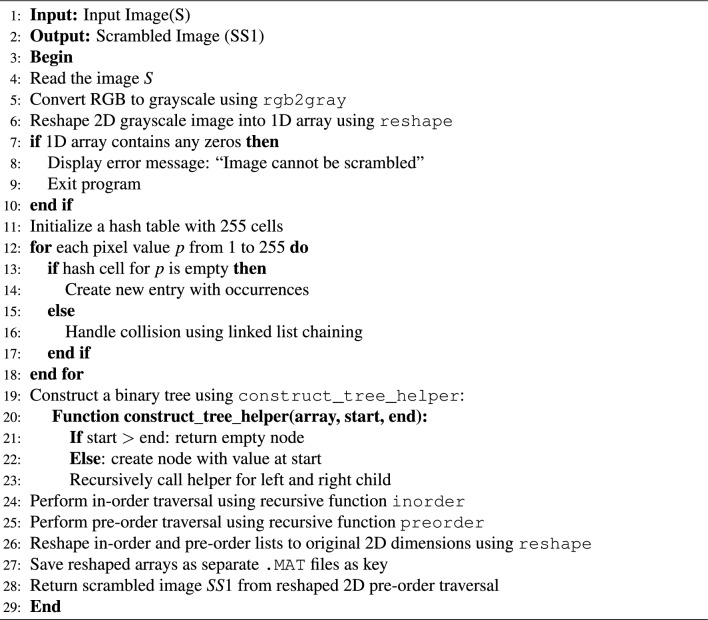
Proposed Image Scrambling Algorithm.

**Fig. 6:**
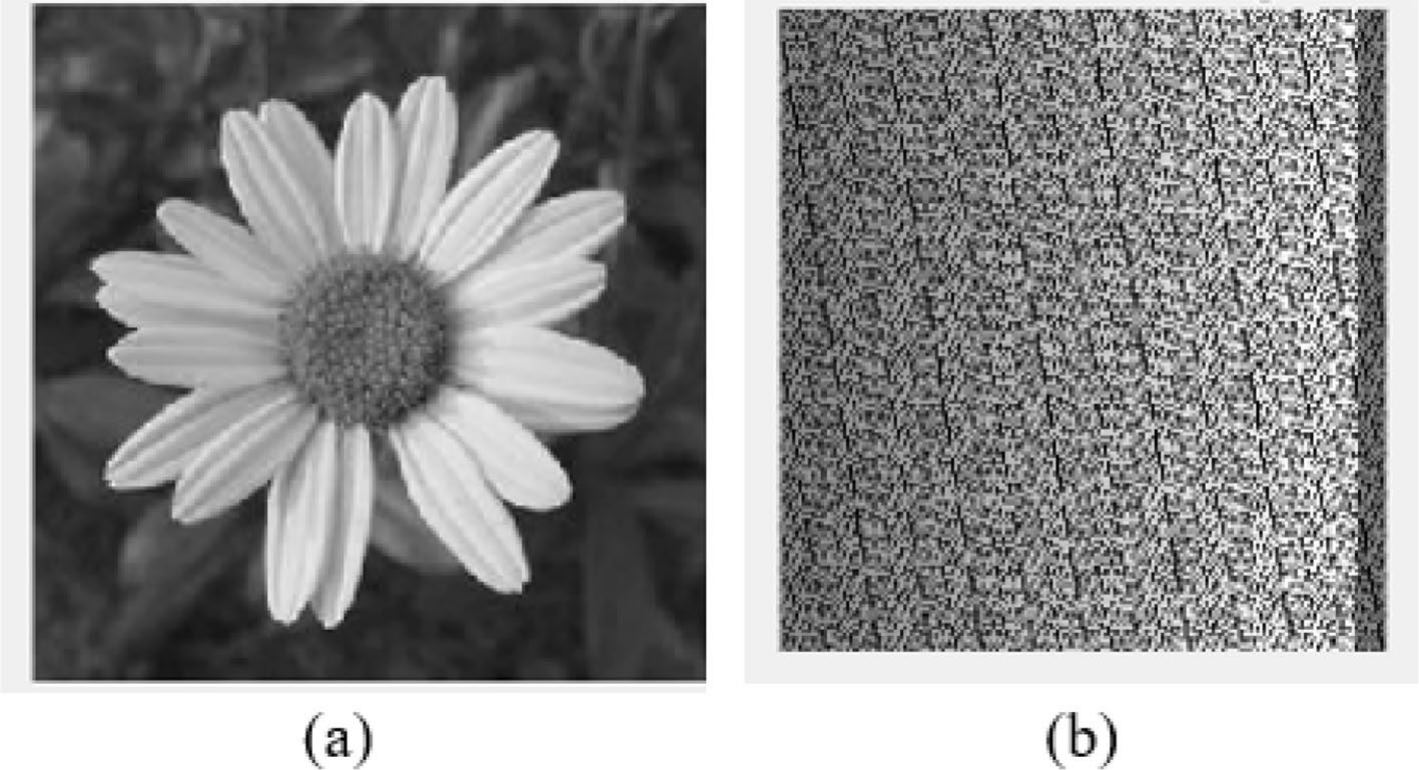
Represent the outcome of proposed scrambling method (**a**) Original Image (S); (**b**) Processed Scrambled image (SS1).

According to the Fig. 6. , the proposed scrambling method applied on image Flower, size of 250 x 250. The image is not containing any zero-pixel value. The outcome 6(b), represent the image is properly scrambled and having PSNR 3.73 dB and MSE 27559.04, which shows that image is properly scrambled and differ from the original input image. According to^23^, if the PSNR is lesser and MSE is high the image is properly scrambled.

### Proposed descrambling method

**Fig. 7:**
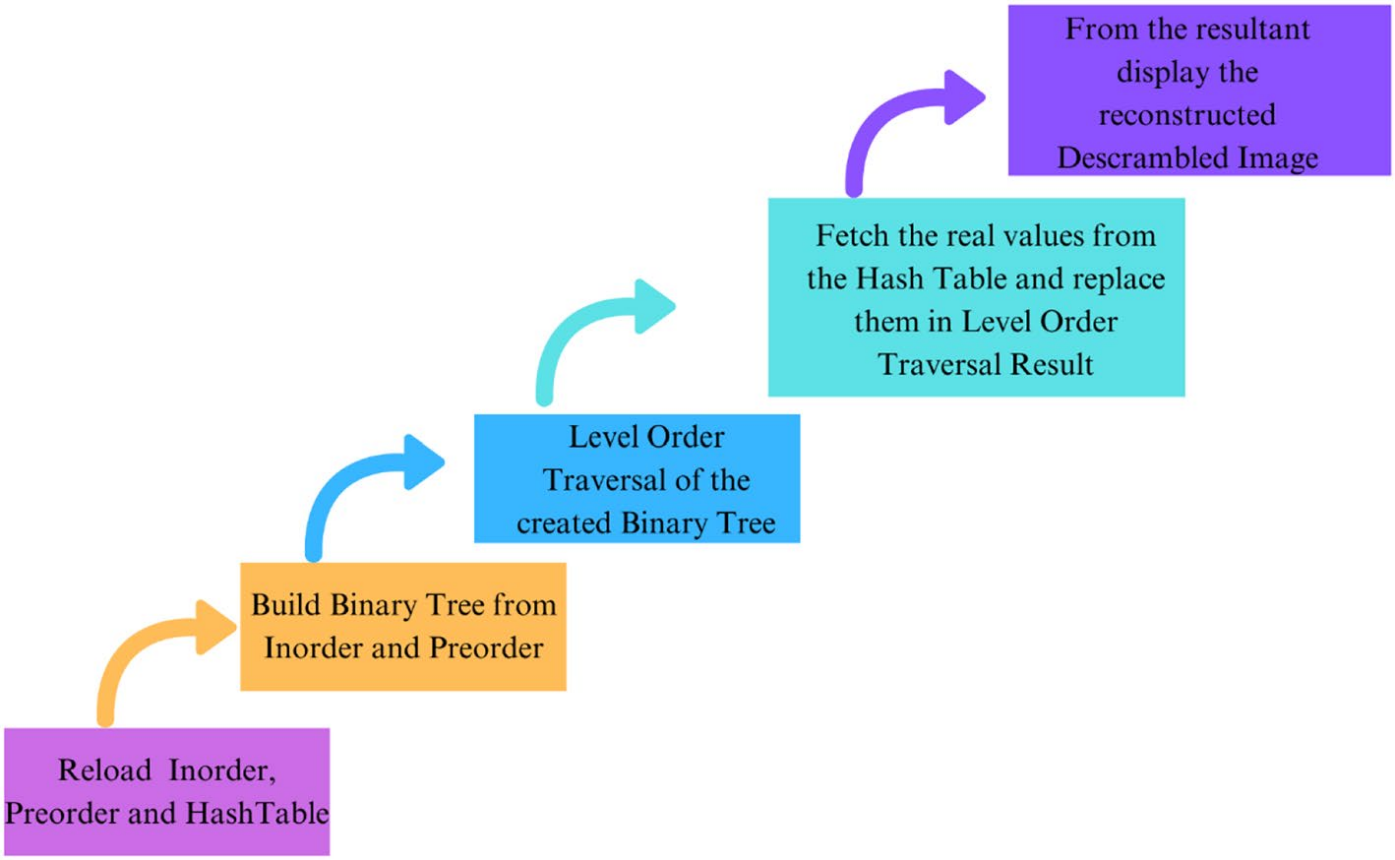
Proposed model for descrambling.

The proposed descrambling method divides the descrambling process into different substages. Starting with loading the in-order traversal, pre-order traversal, and hash table, use them as keys for further processing. Then, building the binary tree from preorder traversal and inorder traversal, followed by level-order traversal on the created binary tree, is performed. Continue with fetching the real values from the hash table associated with the corresponding indices and replacing them in the level-order traversal result. Then, from the resultant array, the descrambled image was reconstructed and displayed. The framework of the proposed descrambling method is shown in Fig. 7 and described in Algorithm ’2’.

**Algorithm 2:**
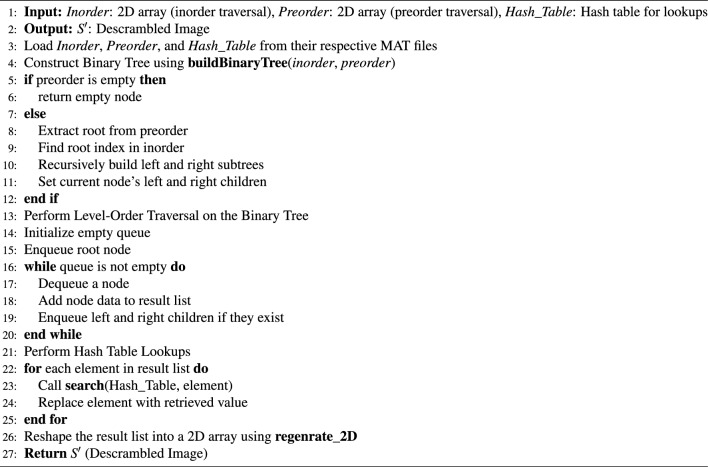
Proposed Image Descrambling Algorithm

**Fig. 8:**
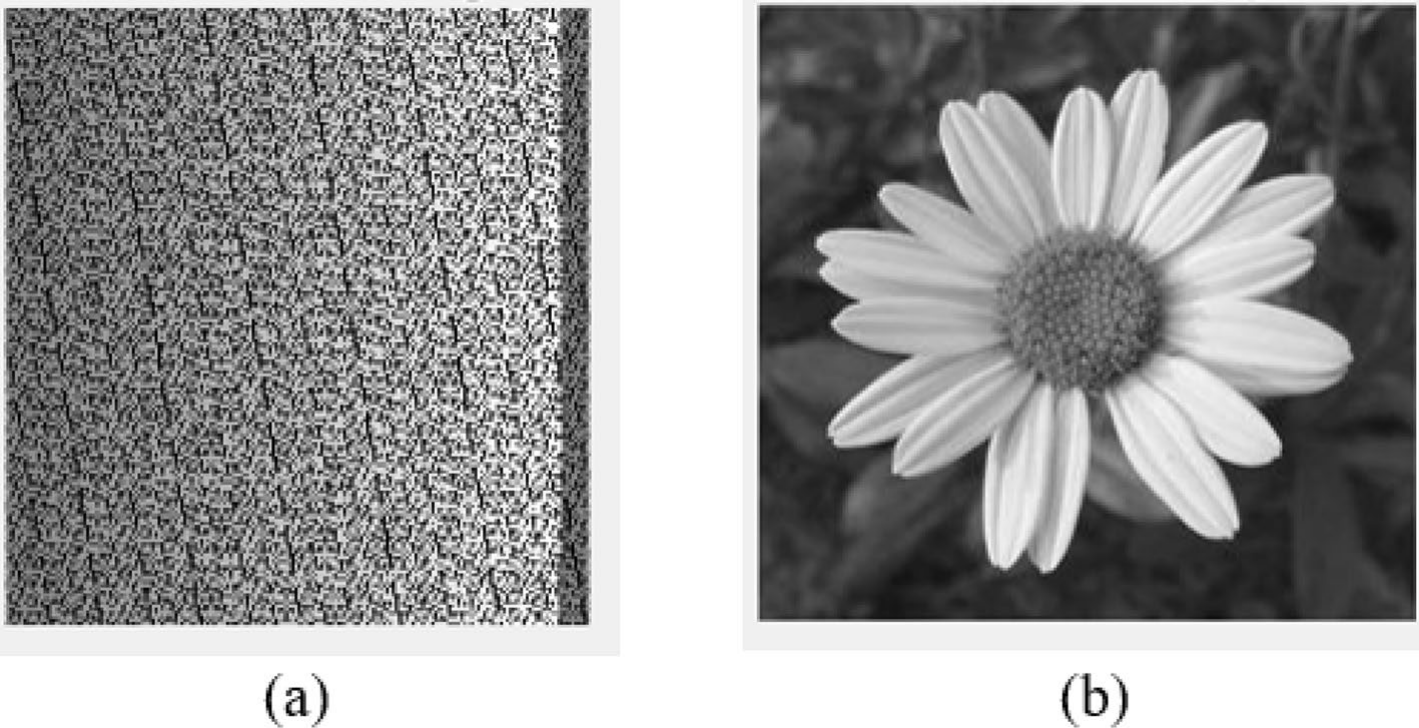
Represent the outcome of proposed Descrambling Method: (**a**) Scrambled Image (SS1); (**b**) Descrambled Image (S’).

According to the Fig. 8., the proposed Descrambling method applied on image Flower, size of 250 x 250.The PSNR and MSE value has been calculated between Descrambled Image and Original Image. The PSNR is Inf dB and MSE is 0, which shows that image is totally Descrambled and same as the original input image. According to^23^, if the PSNR is higher and MSE is lower the image is totally Descrambled.

## Experimental results

This section is divided into two subsections: Subsection 4.1, i.e., “Experimental Setup,” and Subsection 4.2, i.e., “Results.” In Subsection 4.1, a tabulation of the different software and hardware requirements required for this experiment to implement the proposed method has been presented. In the next section, Subsection 4.2 consists of different figures and outputs of different performance measure parameters from the proposed method. The aim of this section is to provide evidence to support the theoretical framework discussed in the previous section and to demonstrate the effectiveness and validity of the proposed method.

### Experimental setup

Table 2, outlines the software and hardware needed to run the program of the proposed method. The software side specifies a compatible operating system (Windows, Linux, or Mac), the programming language (MATLAB), and a specific version of an integrated development environment (IDE) for creating the program. On the hardware side, it details the minimum processor (Intel Core i5), hard drive space (10 GB), and random-access memory (256 MB).

**Table 2:** Requirements of the experiment.

Software requirements	Hardware requirements
Operating System: Windows 10 or above, Linux, or macOS	Processor: Intel Core i5
Programming Language: MATLAB	Hard Disk: Minimum 10 GB
IDE: R2023b Update 6 (23.2.0.2485118)	RAM: 256 MB or more

### Results

For experimental purpose proposed Method has been applied on different images i.e. “Dog.jpg”, “Lenna.jpg”, “Goldhill.jpeg” and “flower.jpg”. As a result, Original Image, Grayscale Image, Scrambled Image and descrambled image have been represented through tabulation in Table 3.

**Fig. 9:**
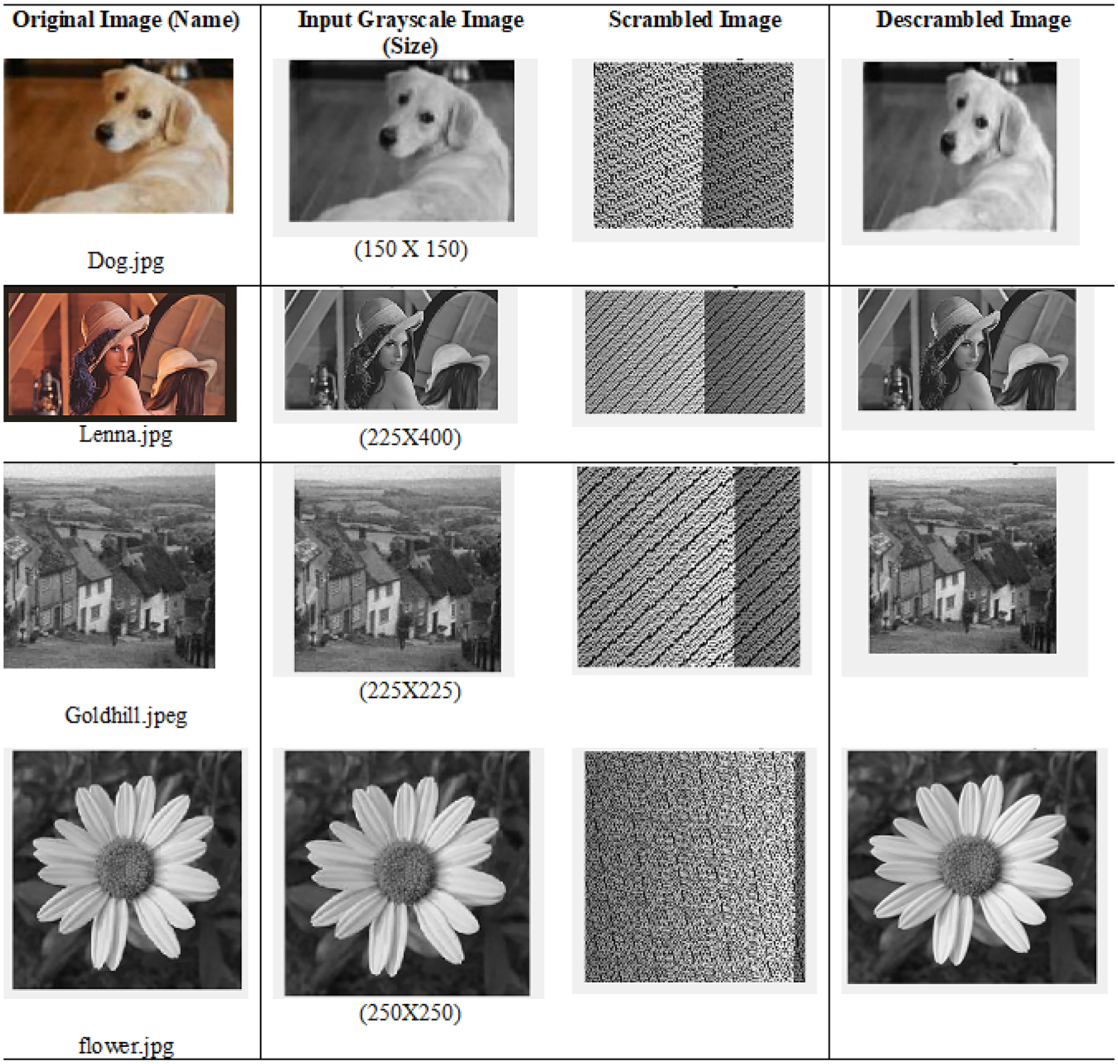
Original image, input grayscale image, scrambled image and descrambled image.

The IQM, which means Image Quality Measurement system, approaches the development of image processing algorithms. In prediction of the processed image performance, so performed, IQM can take place. Quality of the Imagery is defined as the attribute of an image that assesses the quality of services in a processed image by comparing it with an optimal image.

People often use imaging systems to look at pictures, so judging the quality of images by asking people is usually seen as a reliable way to do it. But in situations where things need to be done quickly, like in real-time applications, using people to judge isn’t practical because it is complicated and hard to set up. That is why, in recent years, more people have been using objective methods to assess image quality. The quality assessment parameters details are discussed in this section.

#### Peak signal to noise ratio (PSNR)

Peak Signal-to-Noise Ratio (PSNR)^24^ is a standard way to measure how good a reconstructed image or video looks compared to the original. It helps us understand how much noise is added during processes like compressing, sending, or securing the data. A higher PSNR means better quality, with less noise. PSNR shows the ratio between the strongest part of the signal and the noise that messes it up, usually measured on a scale called decibels. PSNR can be calculated like this:

16 $$\begin{aligned} \text {PSNR (dB)} = 10 \log _{10} \left( \frac{255^2}{\text {MSE}} \right) \end{aligned}$$

#### Mean squared error (MSE)

Mean Squared Error (MSE)^24^ in image encryption is usually used to measure how much the original image and the encrypted image differ. In image encryption, MSE helps us see how much the encryption process changes the image. This ensures that the encrypted image is very different from the original one, which makes it more secure. The formula for MSE is:

17 $$\begin{aligned} \text {MSE} = \frac{1}{mn} \sum _{i=1}^{m} \sum _{j=1}^{n} \left[ I(i,j) - K(i,j) \right] ^2 \end{aligned}$$

Where:

1. I(i,j) represents the pixel intensity of the original image at position (i,j)
2. K(i,j) represents the pixel intensity of the encrypted image at position (i,j)
3. m and n are the dimensions (rows and columns) of the image.

A high MSE value in image encryption means there’s a big difference between the original and encrypted images, which is good for strong encryption. On the other hand, a lower MSE suggests that the encrypted image is too similar to the original, which could weaken security.

#### Normalized cross correlation (NCC)

Normalized Cross-Correlation (NCC)^24^ is commonly used in image processing, especially for finding patterns in images, but it’s also useful for checking how well an image encryption method works. NCC helps measure how similar the original image is to the encrypted one. For a good encryption method, this similarity should be very small, meaning the encrypted image looks very different from the original.

NCC is calculated using this formula:

18 $$\begin{aligned} \text {NCC} = \sum _{i=1}^{m} \sum _{j=1}^{n} \frac{A_{ij} \times B_{ij}}{A_{ij}^2} \end{aligned}$$

An NCC close to zero between the original and encrypted image, for a secure encryption scheme, indicates that the two images are uncorrelated. Hence, a high value of the NCC can indicate poor encryption or, in other words, too much information is retained by the encrypted image from the original image.

#### Average difference (AD)

Average Difference-AD^24^ is a statistical method of analysis for security in the field of image encryption techniques. It denotes how much the pixel values of an original image differ on average from that of the encrypted image. The high value of AD states that the encryption has changed the image significantly, which is quite desirable in strong encryption schemes.

The formula for the calculation of Average Difference (AD) is:

19 $$\begin{aligned} \text {AD} = \frac{1}{mn} \sum _{i=1}^{m} \sum _{j=1}^{n} \left( P_0(i,j) - P_e(i,j) \right) \end{aligned}$$

Where:

1. m denotes the number of rows and n denotes columns.
2. $\hbox {P}\_{0}$ (i,j) represents the pixel value of the original image at a position (i,j)
3. $\hbox {P}\_{\textrm{e}}$ (i,j) is the pixel value at position (i,j) from the encrypted image.
4. The sum of the absolute difference between every original and encrypted pixel value is averaged.

#### Structural content (SC)

Republished from Nat Commun 10, 2864 (2019) Full size imageStructural Content (SC)^24^ is standard metric for quality assessment in Image Processing. It verifies whether structural content of the original image and its corrupted version are similar. It works really well when two images have different level of detail and structure like in this case.

We define the Metric as follows: Structural Content (SC)

20 $$\begin{aligned} \text {SC} = \frac{\sum _{i=1}^{m} \sum _{j=1}^{n} P_0(i,j)^2}{\sum _{i=1}^{m} \sum _{j=1}^{n} P_e(i,j)^2} \end{aligned}$$

Understanding Structural Content (SC):

1. SC = 1: Shows that the images are exactly the same.
2. SC > 1: Means the changed image has less structure compared to the original.
3. SC < 1: Means the changed image has more structure than the original.

SC is very helpful in tasks like compressing images, making them better, and reducing noise, where keeping the original image’s structure is important for quality. But it might not notice small changes in textures or fine details as well as other measures like SSIM (Structural Similarity Index).

#### Maximum difference (MD)

Maximum Difference (MD)^24^, in image processing, is a measure indicating the maximum difference between two images with respect to pixel intensities. It is used in comparing the similarity or difference between a reference image and some processed or otherwise altered version of it. Maximum Difference looks for corresponding pixels between two images and calculates the maximum absolute difference between their intensities.

Calculation:

21 $$\begin{aligned} \text {MD} = \max \left| I_1(x, y) - I_2(x, y) \right| \end{aligned}$$

Where:

$\hbox {I}\_{1}$ (x, y) and $\hbox {I}\_{2}$ (x, y) represent the pixel intensities at the location (x, y) in two images.

The difference is calculated for each pixel, and the maximum value is selected.

Let’s take an example:

Original Image ($$I_1$$):

$$\begin{aligned} I_1 = \begin{bmatrix} 100 & 102 & 104 \\ 101 & 103 & 105 \\ 102 & 104 & 106 \\ \end{bmatrix} \end{aligned}$$

Processed Image ($$I_2$$):

$$\begin{aligned} I_2 = \begin{bmatrix} 98 & 101 & 103 \\ 100 & 102 & 104 \\ 101 & 103 & 105 \\ \end{bmatrix} \end{aligned}$$

To find the MD, we calculate the absolute differences for each pixel and then find the maximum value:

$$\begin{aligned} \begin{aligned} |100 - 98|&= 2 \\ |102 - 101|&= 1 \\ |104 - 103|&= 1 \\ |101 - 100|&= 1 \\ |103 - 102|&= 1 \\ |105 - 104|&= 1 \\ |102 - 101|&= 1 \\ |104 - 103|&= 1 \\ |106 - 105|&= 1 \\ \end{aligned} \end{aligned}$$

The maximum difference here is ’2’, so (MD = 2).

#### Correlation coefficient

In image processing, the correlation coefficient^8^ is a tool used to measure how similar two images are. It’s especially helpful for tasks like finding a pattern in an image, aligning images correctly, and checking how well an image has been rebuilt. The correlation coefficient, usually represented by $\rho$ or r, is a statistical tool that shows how strongly two variables, like two images, are connected in a straight line. In image processing, this helps us see how similar two images are.

For two images $I_1$ and $I_2$, the correlation coefficient can be calculated as:

22 $$\begin{aligned} \rho = \frac{\textrm{Cov}(I_1, I_2)}{\sigma _{I_1} \cdot \sigma _{I_2}} \end{aligned}$$

where,

$\textrm{Cov}(I_1, I_2)$ is the covariance between the two images, and

$\sigma _{I_1}$ and $\sigma _{I_2}$ are the standard deviations of the two images.

#### Number of pixels change rate (NPCR)

NPCR (Number of Pixels Change Rate) is a widely used metric to assess how well encryption or picture scrambling works^25^. It measures the proportion of pixels that differ between two images, typically the original and the encrypted or scrambled version.

23 $$\begin{aligned} \text {NPCR} = \frac{\sum _{i=1}^{M}\sum _{j=1}^{N} D(i,j)}{M \times N} \times 100\% \end{aligned}$$

where

$$D(i,j) = {\left\{ \begin{array}{ll} 0, & \text {if } I_1(i,j) = I_2(i,j) \\ 1, & \text {if } I_1(i,j) \ne I_2(i,j) \end{array}\right. }$$

Here, $I_1$ is the original image, $I_2$ is the scrambled/encrypted image, and $M \times N$ is the total number of pixels.

#### Unified average changing intensity (UACI)

Another common way to compare the average intensity difference between two images is UACI (Unified Average Changing Intensity)^26^. This is usually done between an original image and a scrambled or encrypted image. It works with NPCR by measuring changes in pixel intensity, not just if a pixel changed.

24 $$\begin{aligned} \text {UACI} = \frac{1}{M \times N} \sum _{i=1}^{M} \sum _{j=1}^{N} \frac{|I_1(i,j) - I_2(i,j)|}{255} \times 100\% \end{aligned}$$

Here, $I_1$ is the original image, $I_2$ is the scrambled/encrypted image, and $M \times N$ is the total number of pixels. The division by 255 normalizes the pixel intensity difference for 8-bit images.

**Table 3:** Results of different parameters using proposed scrambling method.

Original Image (Name)	PSNR (dB)	MSE	NCC	AD	SC	MD	NPCR	UACI
Dog.jpg	4.51	23021.37	0.71	0.37	0.99	177	100%	17.13%
Lenna.jpg	3.87	26666.74	0.26	0.06	1.00	140	100%	69.07%
Goldhill.jpeg	4.54	22881.51	0.66	0.13	1.00	222	100%	38.74%
flower.jpg	3.73	27559.04	0.47	0.10	1.00	196	100%	47.90%

Table 3 and Fig. 9 shows how well the new scrambling method works on four different pictures: Dog.jpg, Lenna.jpg, Goldhill.jpeg, and flower.jpg. To figure out which picture is scrambled the best, we need to look at several important measurements: PSNR (Peak Signal-to-Noise Ratio), MSE (Mean Squared Error), NCC (Normalized Cross-Correlation), AD (Absolute Difference), SC (Structural Content), MD (Maximum Difference), NPCR ( Number of Pixels Change Rate), and Unified Average Changing Intensity (UACI).

**Table 4:** Statistical summary of image quality metrics using the proposed scrambling method.

Metric	Mean	Standard Deviation
PSNR	4.1625	0.4226
MSE	25032.1650	2430.7438
NCC	0.5250	0.2047
AD	0.1650	0.1396
SC	0.9975	0.0050
MD	183.7500	34.5097
NPCR	100.0000	0.0000
UACI	43.71	19.87

A low PSNR between the original and scrambled data confirms that scrambling is effective at concealing the original content, while a high PSNR upon descrambling confirms that the original data remains intact and is accurately recoverable. PSNR and MSE, these measures are connected in a way where one increases as the other decreases. A higher PSNR means better image quality, while a higher MSE means more errors. Among the images, “Goldhill.jpeg” has the highest PSNR (4.54 dB) and the lowest MSE (22881.51), showing it keeps the most quality after being changed. On the other hand, “flower.jpg” has the lowest PSNR (3.73 dB) and the highest MSE (27559.04), meaning it is the most distorted.

NCC is a way to measure how similar the original image is to the scrambled one. A smaller NCC number means the image is more scrambled. “Lenna.jpg” has the smallest NCC value (0.26), which shows it is the most scrambled, making it very different from the original. “Dog.jpg” has the biggest NCC value (0.71), meaning it is less scrambled and still looks a lot like the original image.

AD (Average Difference): This measure calculates the average difference between the original and scrambled images. A higher AD value means a bigger difference, which suggests better scrambling. According to the table, “Dog.jpg” has the highest AD (0.37), with “Goldhill.jpeg” coming next (0.13). This shows that “Dog.jpg” is more effectively scrambled in terms of average pixel difference compared to the other images.

SC (Structural Content) measures how similar the structure of the original image is to the scrambled one. A lower SC value means the structure is less similar, which suggests better scrambling. In the table, “Dog.jpg” has an SC value of 0.99, which is slightly less than 1. This shows it is more effectively scrambled in terms of structure compared to the other images, which all have an SC value of 1.

MD reveals the greatest difference in pixel intensity between the original and scrambled images. A higher MD means a bigger maximum change in corresponding pixels, which suggests better scrambling. Goldhill.jpeg has the highest MD value (222), indicating a stronger scrambling effect in some areas, while Lenna.jpg has the lowest MD (140).

Finally, The proposed scrambling method achieves an NPCR of 100% across all tested images, indicating that every pixel changes after scrambling, demonstrating strong resistance against differential attacks. The UACI values are also very high (ranging from 17.13% to 69.07%), showing substantial intensity changes between the original and scrambled images (^27–29^), which confirms the effectiveness of the scrambling at the pixel level.

Table 4 presents the summary statistics of the tested images using the proposed scrambling method. The analysis indicates that a lower average PSNR (mean $\approx 4.16 dB$) and a relatively high MSE (mean $\approx 25,032$) confirm effective scrambling. The low average NCC ($\approx 0.525$) and AD ($\approx 0.165$) suggest minimal similarity between original and scrambled images. The SC value remains close to 1, while the MD shows a moderate variation ($\sim$ 183). Additionally, the NPCR and UACI values demonstrate high randomness, indicating a strong decorrelation between the original and scrambled images.

**Table 5:** Results of different parameters using proposed descrambling method.

Original Image (Name)	PSNR (dB)	MSE	NCC	AD	SC	MD
Dog.jpg	Inf	0	1	0	1	0
Lenna.jpg	Inf	0	1	0	1	0
Goldhill.jpeg	Inf	0	1	0	1	0
flower.jpg	Inf	0	1	0	1	0

Table 5 and Fig. 9 shows the outcomes of using the suggested descrambling technique on four images: “Dog.jpg”, “Lenna.jpg”, “Goldhill.jpeg”, and “Flower.jpg”. The results show that the descrambling process works very well, as all images are fully recovered to their original state. The Peak Signal-to-Noise Ratio (PSNR) for all images is infinite (Inf), which means there is no noise or loss in the recovered images. Also, the Mean Squared Error (MSE) is 0, meaning there is no mistake or difference between the original and descrambled images.

The Normalized Cross-Correlation (NCC) is 1 for all images, showing that the descrambled images are exactly the same as their original versions in terms of pixel connection. Moreover, the Absolute Difference (AD) is 0, meaning there are no pixel-level differences between the original and descrambled images. The Structural Content (SC) for all images is 1, confirming that the structure of the recovered images is identical to that of the original images.

## Comparative study

This paper proposed a new image scrambling method which uses dynamic data structures for encrypting the image. Table 6 compares different methods of scrambling image pixels to evaluate their effectiveness. The values for other scrambling methods are taken directly from Reference^30^ for comparative purposes. We use a common test image called Lenna.jpg. Mixing up the pixels is important for keeping images safe and private by making it hard to see the original picture. We look at several well-known methods and also our new method to see which one does the best job.

The comparison involves various scrambling methods such as Arnold’s Transformation, Fibonacci Transformation, Gray Code Bit Plane, 2D Mapping, Row and Column Shifting, and Row and Column Shifting with Bit Plane Scrambling. Each of these techniques is evaluated against a novel method aimed at improving the scrambling process for increased security and effectiveness.

We assess the performance of these methods using specific parameters, which include Horizontal Correlation (HC), Vertical Correlation (VC), Diagonal Correlation (DC), General Correlation (Corr), and Entropy. These parameters serve as common metrics employed to evaluate the efficacy of scrambling techniques. Correlation measurements (Corr, HC, VC, DC) determine the correlation among adjacent pixels, where a value nearing zero implies superior scrambling effectiveness by diminishing pixel value predictability. On the other hand, Entropy gauges the randomness present in the scrambled image, aiming for an optimal value around 8 for an 8-bit grayscale image to achieve maximum randomness.

The new scrambling method has a correlation value very close to zero (-0.0002), which means it does a good job of reducing the connection between the scrambled image and the original. On the other hand, methods like Arnold’s transformation (2.4868) and Fibonacci transformation (3.1170) have much higher correlation values, showing that the scrambled image still has some link to the original. Other methods, like Gray Code Bit Plane and 2D Mapping, also have low correlation, but the new method does better than most by getting even closer to zero, which is perfect for making the image less predictable and more random.

**Table 6:** Comparative analysis between different scrambling methods and proposed scrambling method for Lenna.jpg^30^.

Parameters	Arnold’s Transformation	Fibonacci Transformation	Gray Code Bit Plane	2D Mapping	Row & Column Shifting	Row & Column Shifting + Bit Plane	Proposed method
Corr	2.4868	3.1170	0.0091	0.0423	0.0139	0.0021	$-$0.0002
HC	0.0327	0.6513	0.1307	0.0626	0.0646	0.0199	0.2855
VC	-0.0875	0.4783	0.1676	0.0660	0.6749	0.0165	0.3336
DC	0.1963	0.2036	0.1323	0.0667	0.0680	0.0158	0.0789
Entropy	7.4455	7.4455	7.4455	0.7691	7.4677	7.9976	7.0505

**Fig. 10:**
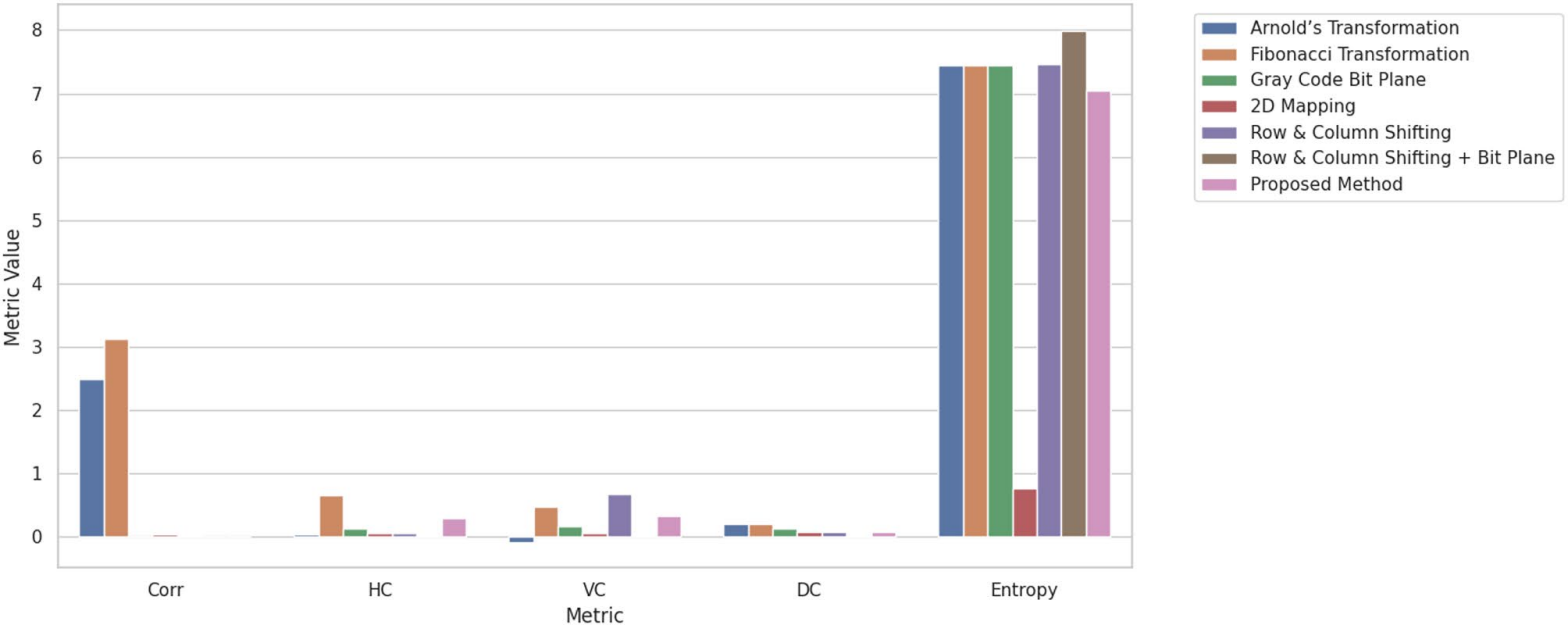
Comparative analysis of scrambling methods for Lenna.jpg.

Figure 10, show that The Proposed Method demonstrates excellent performance in reducing correlation (Corr, HC, VC, DC), indicating effective disruption of pixel relationships — key for security. Entropy^31^ is slightly lower but still acceptable. Overall, the method prioritizes security (low correlation) and structure-aware scrambling, possibly at the slight expense of maximum entropy, which is a reasonable trade-off.Existing scrambling methods often lack reversibility, have high complexity, or offer limited security due to predictable patterns. The proposed approach overcomes these issues by using binary trees and hash tables, ensuring efficient, reversible scrambling with strong security and perfect image reconstruction.

## Conclusion & future scope

In the last few years, the use of digital media transmission has increased. So, providing security to the images is very important so that they cannot be easily accessed by unauthorized third-party users^32^. This paper successfully developed a novel image scrambling and descrambling technique utilizing Dynamic Data Structures like binary trees and hash tables etc. This paper also includes a comparative study between different image encryption techniques. Proposed Scrambling method has been tested over multiple images like “Dog.jpg”, “Lenna.jpg”, “Goldhill.jpeg”, “flower.jpg” and efficiency has been evaluated based on different performance metrices like PSNR^33^, MSE, NCC, SC, AD and MD. From the result section it was observed that Flower.jpg shows the most distortion with the lowest PSNR (3.73 dB) and highest MSE (27559.04). Lenna.jpg has the lowest NCC score (0.26), meaning it is the most scrambled image when compared to the original. Dog.jpg has the highest AD score (0.37) and an SC value of 0.99, showing good scrambling in both average pixel difference and structure. Also, Goldhill.jpeg has the highest MD score (222), indicating the largest differences in pixel intensity, while Lenna.jpg has the lowest MD score (140). These measurements show different levels of how well the images are scrambled. From the comparative study section, it was also observed that the new scrambling method has a correlation value very close to zero (-0.0002), which means it does a good job of reducing the connection between the scrambled image and the original. On the other hand, methods like Arnold’s transformation (2.4868) and Fibonacci transformation (3.1170) have much higher correlation values, showing that the scrambled image still has some link to the original. Other methods, like Gray Code Bit Plane and 2D Mapping, also have low correlation, but the new method does better than most by getting even closer to zero, which is perfect for making the image less predictable and more random.

In future work, we plan to modify the hash function and data structure handling to fully incorporate 0-valued pixels in the scrambling process. This enhancement is expected to improve robustness and ensure better compatibility with practical image datasets.To keep improving the image scrambling algorithm for better speed and efficiency, it’s important to reduce the time it takes to process and use less memory without losing the quality of the scrambled images. Also, using advanced methods like deep learning, specifically convolutional neural networks (CNNs), can help the algorithm learn from data and create more effective scrambling techniques, making the images more secure. Making a simple and easy-to-use interface would help users easily add images, change scrambling settings, see the scrambled results, and use different parts of the algorithm. Lastly, adding strong ways to handle errors will make sure the system can deal with problems like wrong image formats, damaged data, or unexpected user actions, making it more reliable and better for users.

## Data Availability

The image data set used to support the findings of this study is freely available at following datasets links https://www.kaggle.com/datasets/jyotikhandelwal/dynamic-data-structure-based-image-security-method

## References

[1] Venkatesan, C. et al. A novel fuzzy system design for data-adaptive secret key generation and robust audio encryption. *Nonlinear Dyn.*10.1007/s11071-025-11565-7 (2025).

[2] Venkatesh, V. et al. Separable reversible data hiding by vacating room after encryption using encrypted pixel difference. *Sci. Rep.* **15**, 11916. 10.1038/s41598-025-96152-x (2025). 40195368 10.1038/s41598-025-96152-x PMC11977221

[3] Yahya, A. *Steganography Techniques for Digital Images* 1st ed. (Springer, 2018). 10.1007/978-3-319-78597-4.

[4] Abdulla, A. A. Exploiting similarities between secret and cover images for improved embedding efficiency and security in digital steganography, Ph.D. dissertation, Dept. of Applied Computing, Univ. of Buckingham, United Kingdom (2015).

[5] Patel, R. & Bhatt, P. A review paper on digital watermarking and its techniques. *Int. J. Comput. Appl.* **110**, 10–13. 10.5120/19279-0692 (2015).

[6] Mondal, B., Biswas, N. & Mandal, T. A comparative study on cryptographic image scrambling. *Ann. Comput. Sci. Inf. Syst.* **10**, 261–268 (2017).

[7] Agrawal, P., Bhowmick, O. & Prince, Y. Comparative analysis of various steganographic techniques. *Turk. J. Comput. Math. Educ.* **12**(11), 5018–5025 (2021).

[8] Agarwal, S. A review of image scrambling technique using chaotic maps. *Int. J. Eng. Technol. Innov.* **8**(2), 77–98 (2018).

[9] Setiadi, D. R. et al. A comparative study of image cryptographic method. In *Proc. 5th Int. Conf. Inf. Technol. Comput. Electr. Eng. (ICITACEE)* (2018). 10.1109/ICITACEE.2018.8576907.

[10] Surse, N. M. & Vinayakray-Jani, P. A comparative study on recent image steganography techniques based on DWT. In *Proc. Int. Conf. Wireless Commun., Signal Process. Netw. (WiSPNET)*, vol. 1 1308–1314. 10.1109/WiSPNET.2017.8299975 (2017).

[11] Yadav, A. S. & Kumar, S. Comparative analysis of digital image watermarking based on DCT, DWT and SVD with image scrambling technique for information security. In *Proc. Int. Conf. Comput. Characterizat. Techn. Eng. Sci. (CCTES)*, vol. 4 89–93. 10.1109/CCTES.2018.8674135 (2018).

[12] Kabi, K. K., Pradhan, C., Saha, B. J. & Bisoi, A. K. Comparative study of image encryption using 2D chaotic map, In *Proc. Int. Conf. Inf. Syst. Comput. Netw. (ISCON)* **4**, 105–108. 10.1109/ICISCON.2014.6965227 (2014).

[13] Amirtharajan, R., Akila, R. & Deepikachowdavarapu, P. A comparative analysis of image steganography. *Int. J. Comput. Appl.* **2**, 41–47. 10.5120/644-900 (2010).

[14] Pawar, S. S. & Nandusekar, S. Improvised image scrambling technique with shuffling of pixel values and position. In *Proc. Int. Conf. Commun. Electron. Syst. (ICCES)* **31**, 1–5. 10.1109/CESYS.2016.7889836 (2016).

[15] Jeelani, Z. & Qadir, F. A comparative study of cellular automata-based digital image scrambling techniques. *Evol. Syst.* **12**, 359–375. 10.1007/s12530-020-09326-5 (2020).

[16] Turan, M., Gökçay, E. & Tora, H. An unrestricted Arnold’s Cat Map transformation. *Multimed. Tools Appl.* **83**, 70921–70935. 10.1007/s11042-024-18411-9 (2024).

[17] Mishra, M. Image encryption using Fibonacci-Lucas Transformation. *Int. J. Cryptogr. Inf. Secur.* **2**, 131–141. 10.5121/ijcis.2012.2312 (2012).

[18] Zhou, R. G., Sun, Y. J. & Fan, P. Quantum image gray-code and bit-plane scrambling. *Quantum Inf. Process.* **14**, 1717–1734. 10.1007/s11128-015-0964-6 (2015).

[19] Chen, C. F. & Lucas, A. Finite speed of quantum scrambling with long-range interactions. *Phys. Rev. Lett.*10.1103/PhysRevLett.123.250605 (2019). 31922813 10.1103/PhysRevLett.123.250605

[20] Bashir, Z., Iqbal, N. & Hanif, M. A novel gray scale image encryption scheme based on pixels’ swapping operations. *Multimedia Tools Appl.* **80**, 1029–1054. 10.1007/s11042-020-09695-8 (2020).

[21] Song, F. Y., Xu, G.-B., Wang, H.-K. & Jiang, D.-H. A quantum image encryption algorithm based on chaotic system and Rubik’s cube principle. *Quantum Inf. Process.*10.1007/s11128-024-04492-w (2024).

[22] Ponnambalam, M., Ponnambalam, M. & Jamal, S. S. A robust color image encryption scheme with complex whirl wind spiral chaotic system and quadrant-wise pixel permutation. *Phys. Scr.* **99**(10), 105239. 10.1088/1402-4896/ad7075 (2024).

[23] Sara, U., Akter, M. & Uddin, M. S. Image quality assessment through FSIM, SSIM, MSE and PSNR–a comparative study. *J. Comput. Commun.* **7**, 8–18. 10.4236/jcc.2019.73002 (2019).

[24] Memon, F., Unar, M. A. & Memon, S. Image quality assessment for performance evaluation of focus measure operators (2016).

[25] Ponnambalam, M. et al. Hybrid inter woven scrambling with spiral shell 3D hyperchaotic diffusion for secure color image encryption. *Nonlinear Dyn.*10.1007/s11071-025-11460-1 (2025).

[26] Devi, C. S. & Amirtharajan, R. A novel 2D MTMHM based key generation for enhanced security in medical image communication. *Sci. Rep.* **15**(1), 25411. 10.1038/s41598-025-10485-1 (2025). 40659745 10.1038/s41598-025-10485-1 PMC12259849

[27] Banu, A. & Amirtharajan, R. A robust medical image encryption in dual domain: Chaos-DNA-IWT combined approach. *Med. Biol. Eng. Comput.* **58**, 1445–1458. 10.1007/s11517-020-02178-w (2020). 32319032 10.1007/s11517-020-02178-w

[28] Ravichandran, D., Praveenkumar, P., Rayappan, J. B. B. & Amirtharajan, R. DNA chaos blend to secure medical privacy. *IEEE Trans. Nanobiosci.* **16**(8), 850–858. 10.1109/TNB.2017.2780881 (2017). 10.1109/TNB.2017.2780881 29364129

[29] Ravichandran, D., Praveenkumar, P., Rayappan, J. B. B. & Amirtharajan, R. Chaos-based crossover and mutation for securing DICOM image. *Comput. Biol. Med.* **72**, 170–184. 10.1016/j.compbiomed.2016.03.020 (2016). 27046666 10.1016/j.compbiomed.2016.03.020

[30] Mondal, B. Cryptographic image scrambling techniques. In *Cryptographic and Information Security* 37–65 (2018). 10.1201/9780429435461-2.

[31] Nithya, C. et al. Secure gray image sharing framework with adaptive key generation using image digest. *Sci. Rep.* **15**, 8854. 10.1038/s41598-025-92752-9 (2025). 40087325 10.1038/s41598-025-92752-9 PMC11909240

[32] Abdulla, A. A., Sellahewa, H. & Jassim, S. A. Stego quality enhancement by message size reduction and fibonacci bit-plane mapping. In *Security Standardisation Research (SSR 2014). Lecture Notes in Computer Science* (eds Chen, L. & Mitchell, C.) vol. 8893 149–164 (Springer, 2014). 10.1007/978-3-319-14054-4_10.

[33] Ravichandran, D. et al. An efficient medical data encryption scheme using selective shuffling and inter-intra pixel diffusion IoT-enabled secure E-healthcare framework. *Sci. Rep.* **15**, 4143. 10.1038/s41598-025-85539-5 (2025). 39900990 10.1038/s41598-025-85539-5 PMC11790928

